# Complete Genome Assembly of *Corynebacterium* sp. Strain ATCC 6931

**DOI:** 10.1128/genomeA.01074-14

**Published:** 2014-10-23

**Authors:** H. E. Daligault, K. W. Davenport, T. D. Minogue, K. A. Bishop-Lilly, D. C. Bruce, P. S. Chain, S. R. Coyne, K. G. Frey, J. Jaissle, G. I. Koroleva, J. T. Ladner, P-E. Li, L. Meincke, A. C. Munk, G. F. Palacios, C. L. Redden, S. L. Johnson

**Affiliations:** a Los Alamos National Laboratory (LANL), Los Alamos, New Mexico, USA; b Diagnostic Systems Division (DSD), United States Army Medical Research Institute of Infectious Diseases (USAMRIID), Fort Detrick, Maryland, USA; c Naval Medical Research Center (NMRC-Frederick), Fort Detrick, Maryland, USA; d Henry M. Jackson Foundation, Bethesda, Maryland, USA; e Center for Genome Sciences (CGS), USAMRIID, Fort Detrick, Maryland, USA

## Abstract

The genus *Corynebacterium* is best known for the pathogen *C. diphtheriae*; however, it contains mostly commensal and nonpathogenic, as well as several opportunistic, pathogens. Here, we present the 2.47-Mb scaffolded assembly of the type strain, *Corynebacterium* sp*.* ATCC 6931 (NCTC 1914), as deposited into GenBank under accession number CP008913.

## GENOME ANNOUNCEMENT

The genus *Corynebacterium* includes slow-growing Gram-positive rod-shaped chemo-organotrophs. The genus is named for their “knotted rod” (Greek *corönë*) cellular shape and was first described in the 1890s (*Corynebacterium diphtheriae*, the causative agent of diphtheria). Most described species are commensals or soil/freshwater dwelling; however, there are several species known to be opportunistic pathogens, especially of the immunocompromised (1). Here, we present a scaffolded genome assembly of the type strain *Corynebacterium* sp*.* ATCC 6931 (NCTC 1914).

High-quality genomic DNA was extracted from a purified isolate using QIAgen Genome Tip-500 at USAMRIID-DSD. Specifically, a 100-mL bacterial culture was grown to the stationary phase and nucleic acid was extracted per the manufacturer’s recommendations. Sequence data were generated using two separate Illumina technologies (2, 3). We constructed and sequenced two Illumina libraries: standard (unpaired) 100-bp reads (300-fold genome coverage) and a separate long-insert paired-end library (9-fold genome coverage, 7,954 ± 2,596 bp insert). Data from the two libraries were assembled together in Newbler (Roche) and the consensus sequences computationally shredded into 2-kbp overlapping fake reads (shreds). The raw reads were also assembled in Velvet and those consensus sequences computationally shredded into 1.5-kbp overlapping shreds (4). All draft data were then assembled together in ALLPATHS and the consensus sequences were computationally shredded into 10-kbp overlapping shreds (5). We then integrated the Newbler consensus shreds, Velvet consensus shreds, ALLPATHS consensus shreds, and a subset of the long-insert read-pairs using parallel Phrap (High Performance Software). Possible misassemblies were corrected and some gap closure was accomplished with manual editing in Consed (6–8).

Automatic annotation of the *Corynebacterium* sp. ATCC 6931 genome assembly utilized an Ergatis-based workflow at LANL with minor manual curation. The closed, quality-checked, annotated assembly is available in NCBI (CP008913), and the raw data can be provided upon request. Preliminary review of the annotation indicates 26 genes associated with antibiotic and toxic-metal resistance, in addition to several genes associated with a *Mycobacterium* virulence operon (9). Based on a BWA (10) contig classifier and Mega-BLAST (11) to the NCBI nucleotide database, this strain is most closely related to *C. urealyticum* (known to cause antibiotic resistant urinary tract infections) (12); however, further placement efforts are warranted.

### Nucleotide sequence accession number.

The finished genome assembly for *Corynebacterium* sp. ATCC 6931 has been deposited in GenBank under accession number CP008913.
